# A prospective, double-blind, randomized, two-period crossover, multicenter study to evaluate tolerability and patient preference between mirabegron and tolterodine in patients with overactive bladder (PREFER study)

**DOI:** 10.1007/s00192-017-3377-5

**Published:** 2017-06-15

**Authors:** David Staskin, Sender Herschorn, Jonathan Fialkov, Le Mai Tu, Terry Walsh, Carol R. Schermer

**Affiliations:** 1 Tufts University School of Medicine, Boston, MA 02135 USA; 2 Sunnybrook Health Sciences Centre, University of Toronto, Toronto, ON Canada; 3 The Iowa Clinic, West Des Moines, IA USA; 4 University of Sherbrooke, Quebec, Canada; 5 Astellas Pharma US, Northbrook, IL USA

**Keywords:** Crossover study, Mirabegron, Tolterodine, OAB-S Medication Tolerability scale, Patient preference, Anticholinergic side effects

## Abstract

**Introduction and hypothesis:**

The objective of this study was to assess the tolerability and treatment preference in patients with overactive bladder (OAB) treated with mirabegron or tolterodine.

**Methods:**

This was a two-period, 8-week crossover, double-blind, phase IV study (PREFER; NCT02138747) in treatment-naive adults with OAB for 3 months or longer randomized to one of four treatment sequences in a 5:5:1:1 ratio (mirabegron/tolterodine, tolterodine/mirabegron, mirabegron/mirabegron, or tolterodine/tolterodine), separated by a washout period of 2 weeks. The primary endpoint was drug tolerability using the Medication Tolerability scale of the OAB Treatment Satisfaction (OAB-S) questionnaire at end of treatment (EoT). Period-by-treatment interactions were analyzed to determine any effect of drug order. Patient preference, change from baseline in OAB symptoms, and treatment-emergent adverse events (TEAEs) were assessed.

**Results:**

A total of 358 randomized patients completed the OAB-S Medication Tolerability scale questionnaire at one or more visits after the baseline evaluation. The mean (95% CI) OAB-S Medication Tolerability scores were significantly higher (better tolerability) for mirabegron (86.29 [83.50, 89.08]) than for tolterodine (83.40 [80.59, 86.20]; *p* = 0.004). The period-by-treatment interaction was not significant (*p* = 0.955). Improvements in OAB-S Medication Tolerability scores at EoT were more evident in women, patients aged ≥65 years, and in patients without baseline incontinence, and were greater with mirabegron than with tolterodine extended release. There were no significant differences in patient preference or improvements in OAB symptoms. Significant differences in favor of mirabegron were observed for anticholinergic TEAEs (20.4% vs. 27.4%; *p* = 0.042) and specifically for gastrointestinal disorders (14.7% vs. 22.5%; *p* = 0.015).

**Conclusions:**

Tolerability of mirabegron was significantly higher than that of tolterodine, and patient preference and improvements in OAB symptoms were comparable. Both treatments were well tolerated; however, anticholinergic side effects were higher with tolterodine.

**Electronic supplementary material:**

The online version of this article (doi:10.1007/s00192-017-3377-5) contains supplementary material, which is available to authorized users.

## Introduction

Overactive bladder (OAB) is a highly prevalent syndrome defined as urinary urgency, usually accompanied by day-time frequency and nocturia, with or without urinary incontinence, in the absence of urinary tract infection or other obvious pathology [1, 2]. The chronic nature of OAB and its impact on daily activities often results in significantly impaired quality of life (QoL) including psychological/emotional distress, depression, and social isolation [3].

Oral pharmacotherapies, antimuscarinics (e.g., tolterodine) and the β-3-adrenoceptor agonist, mirabegron, have similar efficacy. However, in one of the mirabegron registration trials in which tolterodine was an active control, and in a recent review, a systematic literature review and mixed treatment comparison of multiple randomized clinical trials, the frequency of side effects typical of anticholinergic use was found to be lower with mirabegron than with antimuscarinic agents [4–6]. Dry mouth, the most frequent side effect of antimuscarinics [7], is one of the main reasons patients discontinue treatment [8].

The successful management of OAB requires long-term treatment persistence, which relies on symptom improvement, along with the patient’s adverse event experiences, and whether improvements translate into positive changes in daily routine and psychological wellbeing [9]. Among numerous patient-reported outcomes used to evaluate OAB therapies, the multidimensional concept of patient satisfaction is one of the more important, encompassing efficacy, safety/tolerability and QoL, while also accounting for non-health-related factors such as sociodemographics, physical/psychological status, attitude and treatment expectations [10]. Patient satisfaction is predictive of long-term persistence and may be more sensitive to changes in wellbeing than questionnaires focusing on QoL [11].

The OAB Treatment Satisfaction (OAB-S) questionnaire is a validated instrument consisting of five independent scales related to OAB (control expectations, impact on daily living, control, medication tolerability, and satisfaction with control), and five single-item overall assessments, that have demonstrated satisfactory psychometric performance [12]. Individual components, such as the OAB Medication Tolerability scale, can be evaluated in isolation to focus on specific benefits of treatment [13].

The primary objective of this two-period crossover study (PREFER study; NCT02138747) in patients with OAB was to compare the tolerability of mirabegron and tolterodine extended release (ER), based on the OAB-S questionnaire. Secondary objectives included assessment of patient preference, safety, and changes in bladder diary outcomes.

## Materials and methods

### Study design and participants

**Fig. 1:**
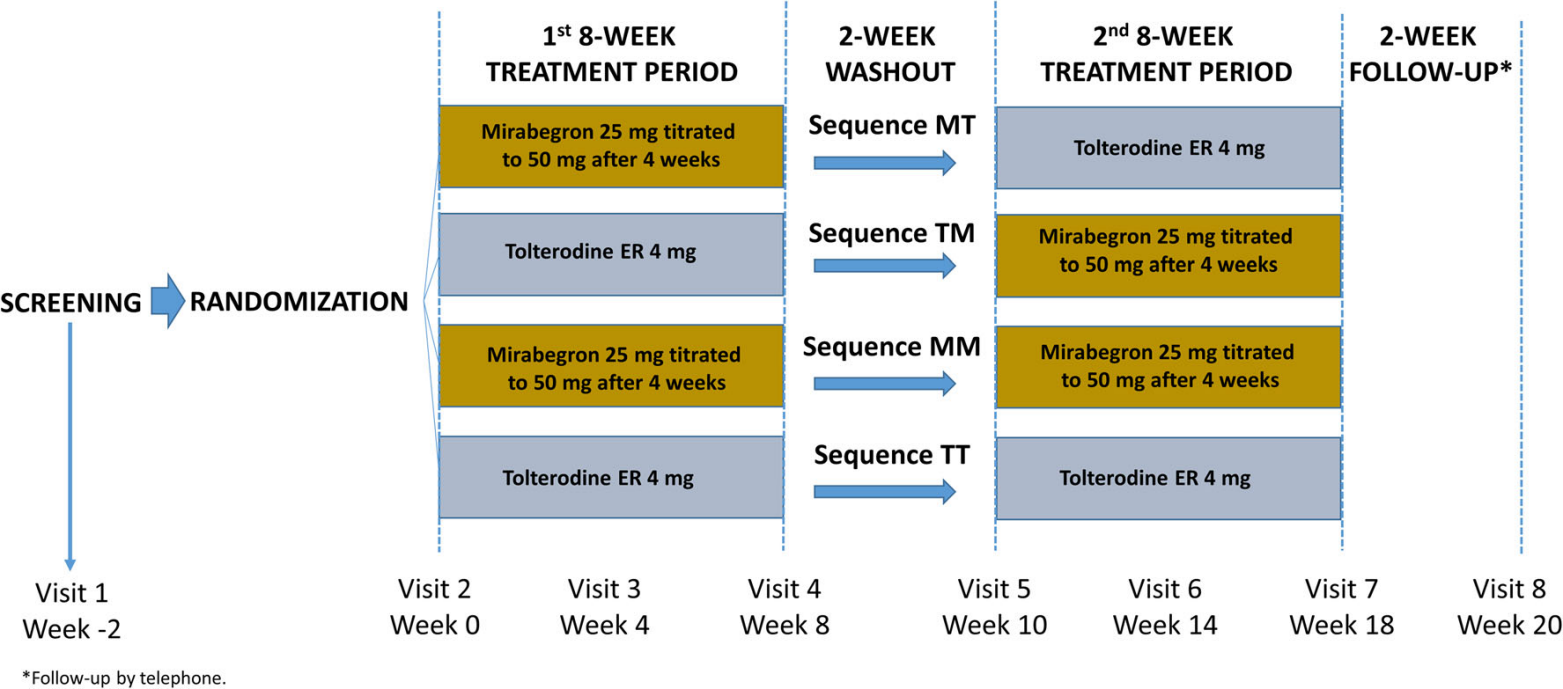
Study design

This prospective, double-blind, active-controlled, higher order (i.e., number of periods/sequences > number of treatments being compared [14]), two-period crossover, phase IV study, was conducted at 36 sites (28 sites in the US and 8 sites in Canada). Treatment-naive adults with OAB for 3 months or longer were randomized to one of the following four treatment sequences in a 5:5:1:1 ratio: mirabegron (M)/tolterodine 4 mg ER (T), T/M, M/M, and T/T; Fig. 1; see Supplementary file 1 Randomization and blinding). Based on a 3-day electronic bladder diary, eligible patients had three or more episodes of urgency over 3 days (Patient Perception of Intensity of Urgency Scale, PPIUS [15], grade 3 or 4) and an average of eight or more micturitions over 24 h at baseline (Supplementary Table 1 Inclusion/exclusion criteria).

After completing the first 8-week treatment period, patients entered a 2-week washout period followed by a second baseline visit during week 10. Patients completed a 3-day bladder diary prior to visits at baseline (week 0/week 10) and weeks 4/14 and 8/18 during double-blind treatment periods. At each follow-up visit in both treatment periods, patients completed the Medication Tolerability scale of the OAB-S questionnaire. At the end of the second treatment period (week 18 or end of treatment, EoT), patients rated their treatment preference and the degree of preference on a five-point Likert scale (strong preference for period 1, mild preference for period 1, no preference, mild preference for period 2, strong preference for period 2). Patients therefore received both mirabegron and tolterodine ER in sequence.

During weeks 4/14 the dose of mirabegron was increased from 25 mg to 50 mg. Patients who discontinued a treatment period were asked to complete a 3-day bladder diary and questionnaires for that period. The total study duration was 22 weeks, including a follow-up phone call 2 weeks after the EoT.

### Efficacy assessments

The primary endpoint was medication tolerability assessed using the Medication Tolerability scale of the OAB-S questionnaire at EoT of each period. The Medication Tolerability scale measures the level of bother associated with six side effects (items) related to OAB medications (constipation, dry mouth, drowsiness, headache, nausea and blurred vision) on a scale of 1 (“bothered a lot”) to 6 (“did not have side effect”) and the final score (0–100; higher score representing better tolerability) calculated as: ([sum of final response values for completed items/number of completed items] − 1) × 20. These side effects are commonly associated with anticholinergics.

Treatment differences were relative to mirabegron in the M/T and T/M sequences (negative difference indicating better tolerability with mirabegron), and relative to period 2 in the M/M and T/T sequences (negative difference indicating better tolerability during period 2). To allow direct comparison of the OAB-S Medication Tolerability scores between mirabegron and tolterodine ER, it was necessary to test for an effect of sequence on the mean OAB-S Medication Tolerability scores by confirming a nonsignificant period-by-treatment interaction (*p* > 0.05).

For the key secondary endpoint, treatment preference was assessed using the five-point Likert scale in patients receiving the M/T and T/M sequences who completed ≥14 days of each treatment period and rated their preference at the end of period 2. Patients were asked to identify one or more of the following reasons for their preference ‘better treatment’, ‘better tolerated’, and ‘other’. At the end of period 2, the investigator was also asked to identify their preferred treatment and degree of preference as ‘mild’ or ‘strong’ on a similar five-point Likert scale.

Other secondary efficacy endpoints assessed at EoT included: mean change from baseline in bladder diary variables of incontinence, micturition frequency, urgency, urgency incontinence, and nocturia. Other secondary analyses included responder analysis based on the percentage of patients achieving zero incontinence episodes and those achieving ≥50% reduction from baseline in incontinence episodes; and the frequency and severity of the six individual components of the OAB-S Medication Tolerability scale. There is no published minimally important difference for the OAB-S; however, a responder was defined a priori as a patient achieving an OAB-S Medication Tolerability scale score of ≥90 out of 100.

Subgroup analyses based on patient age (<65 or ≥65 years), sex, and baseline incontinence (‘wet’ or ‘dry’) were investigated for the OAB-S Medication Tolerability score (a priori) and patient preference (post hoc).

### Safety assessments

The frequency of treatment-emergent adverse events (TEAEs), including those of special interest (e.g., anticholinergic and cardiovascular), are summarized by treatment. Vital signs were assessed at each visit and mean changes from baseline to EoT calculated.

### Statistical analysis

It was planned to screen approximately 450 patients to achieve 360 randomized patients, assuming 20% dropout between screening and randomization. Sample sizes were calculated considering the primary and key secondary efficacy endpoints. For the OAB-S Medication Tolerability score, data were assumed to be normally distributed with a mean difference of 7 between treatments, and a pooled standard deviation (SD) of 20.11. A sample size of 124 patients per M/T and T/M sequence at *α* = 0.05 yielded ≥99% power to detect a mean difference of 7 in the OAB-S Tolerability score between treatments.

For patient preference, 99 patients per M/T and T/M sequence was determined as necessary to detect a 20% difference between mirabegron and tolterodine ER with 80% power and *α* = 0.05 based on the Mainland-Gart test. This assumed that 60% and 40% of patients with a preference, respectively, preferred mirabegron and tolterodine ER; if 20% had no preference, 124 patients per sequence needed to be randomized. Two additional sequences, M/M and T/T (30 patients receiving each), were included to assess potential carry-over effects, enable direct comparison of treatments, and provide unbiased estimates of treatment and carry-over effects.

The full analysis set (FAS) population comprised all randomized patients who received one or more doses of the study medication on a double-blind basis, and completed the OAB-S Medication Tolerability scale questionnaire at one or more post-baseline visits. The FAS-Incontinence (FAS-I) population comprised FAS patients with one or more incontinence episodes at baseline during period 1 who completed one or more bladder diary entries for one or more post-baseline visits during period 1. The safety analysis set (SAF) population comprised all randomized patients who received one or more doses of the study medication on a double-blind basis. The FAS–preference/no preference (FAS-PNP) population comprised all randomized patients who received the study medication on a double-blind basis for 14 days or longer in each period, and completed the patient preference score at the end of period 2.

The OAB-S Medication Tolerability scores were analyzed using analysis of variance (ANOVA) with sequence, period, period-by-treatment interaction, sex and treatment as factors, and patient-within-sequence as a random term. Least squares (LS) mean OAB-S Medication Tolerability scores, two-sided 95% confidence intervals (CI) and *p* values for the mean treatment differences and period-by-treatment interactions were derived from the ANOVA model. In addition, LS mean estimates (95% CI) are displayed by period within sequence and for each treatment. Unadjusted mean (standard error, SE) OAB-S Medication Tolerability scores were analyzed in a FAS subset of patients who completed the OAB-S Medication Tolerability score questionnaire in both treatment periods (complete cases), to determine whether patients who discontinued treatment during period 1 had a lower tolerability score. Preferences of patients in the FAS-PNP population receiving M/T and T/M sequences were analyzed using the Mainland-Gart test, which adjusted for the effect between study periods and excluded patients with no preference. Preferences in the FAS-PNP population including patients with no preference for either period were investigated in a separate analysis. Frequencies are presented for strong preference or physician preference; no statistical testing was performed.

Changes from baseline to EoT for each period in bladder diary variables were analyzed using analysis of covariance (ANCOVA) with sequence, period, period-by-treatment interaction, sex and treatment group as factors, baseline value as a covariate, and patient-within-sequence as a random term. The LS mean estimate and two-sided 95% CI for the mean changes from baseline were derived from the ANCOVA model. The numbers and percentages of patients who selected each component of the OAB-S Medication Tolerability score (constipation, dry mouth, drowsiness, headache, nausea and blurred vision) at the end of each treatment period are presented for the FAS. No statistical testing was performed for the individual components of the OAB-S Medication Tolerability score.

TEAEs are summarized descriptively by system organ class (SOC), preferred term, and treatment; TEAEs reported in both periods of the M/M and T/T sequences were counted once. Vital signs (systolic blood pressure, diastolic blood pressure, and pulse rate) are summarized in terms of mean (SD) by treatment group. For anticholinergic, cardiovascular and urinary retention TEAEs of special interest, *p* values from Fisher’s exact test comparing treatments are presented for the number of patients with one or more TEAEs for each side effect or SOC. These calculations were planned a priori but were not considered in the sample size calculations.

## Results

### Patient demographics and baseline characteristics

**Fig. 2:**
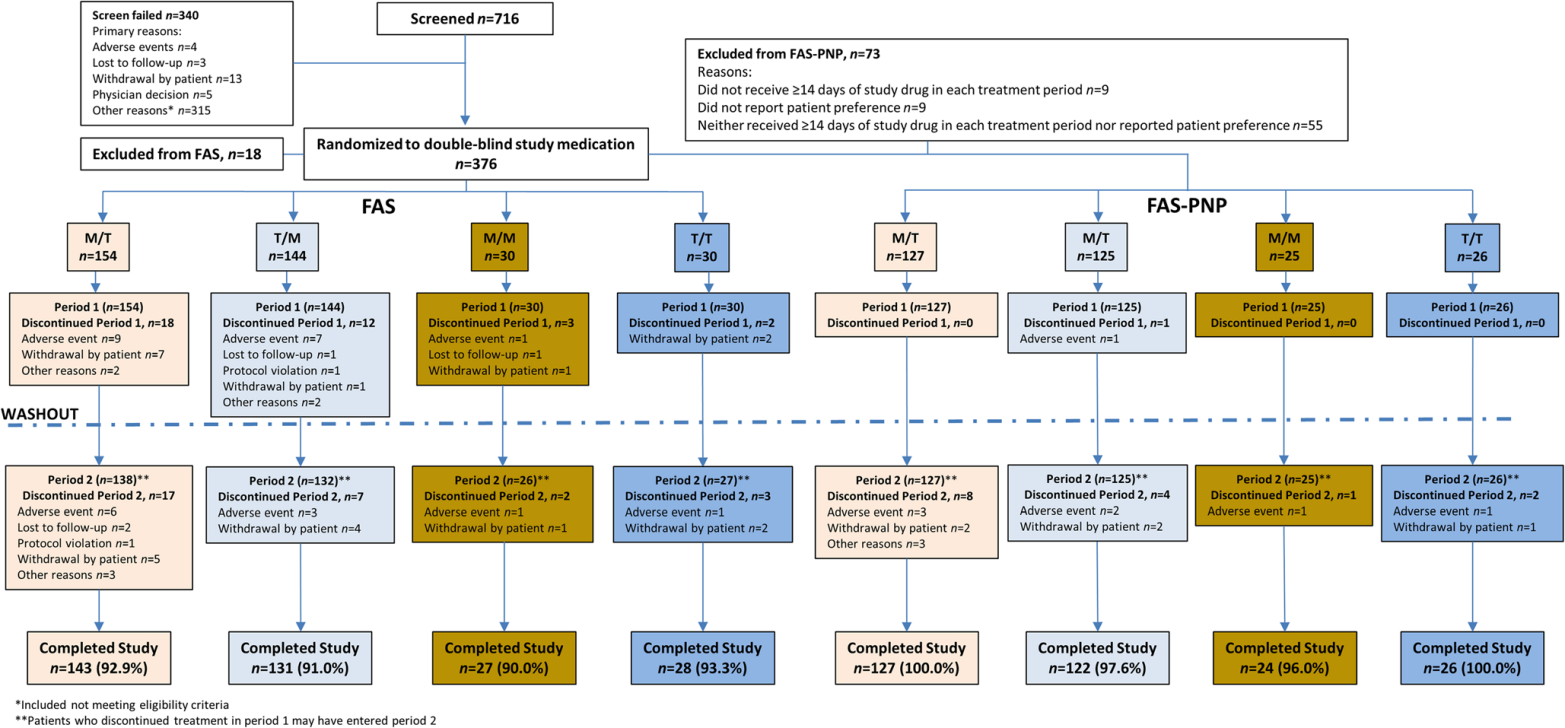
Patient disposition in the full analysis set (*FAS*) and full analysis set–preference/no preference (*FAS-PNP*) populations

A total of 376 patients were randomized: 156 patients received the M/T sequence, 157 the T/M sequence, 31 the M/M sequence, and 32 the T/T sequence. In the FAS, 329 patients (91.9%) completed the study and 29 patients (8.1%) discontinued the study due to withdrawal by patient (13, 3.6%), lost to follow-up (9, 2.5%), and other reasons (7, 2.0%; Fig. 2).

The demographics of the patients receiving the M/T and T/M sequences were comparable, except that there were fewer incontinent patients at baseline, and fewer patients aged ≥65 years who received tolterodine ER in period 1 (Table 1). Overall, patients had moderate-to-severe symptoms of OAB at baseline, i.e., more than four urgency episodes (PPIUS grade 3 or 4) per 24 h, more than ten micturitions per 24 h, and approximately 2.7 incontinence episodes per 24 h.

**Table 1:** Demographics and baseline OAB characteristics of the full analysis set in period 1 of each sequence, and in the total treatment groups

	Period 1				Total	
	M/T (*n* = 154)	T/M (*n* = 144)	M/M (*n* = 30)	T/T (*n* = 30)	Mirabegron (*n* = 316^a^)	Tolterodine (*n* = 310^a^)
Women, *n* (%)	116 (75.3)	108 (75.0)	18 (60.0)	20 (66.7)	232 (73.4)	233 (75.2)
Age (years), mean (SD)	53.5 (14.8)	52.3 (12.6)	59.0 (13.1)	54.9 (14.8)	53.4 (13.9)	53.2 (13.7)
Age group, *n* (%)						
<65 years	117 (76.0)	120 (83.3)	20 (66.7)	21 (70.0)	247 (78.2)	243 (78.4)
≥65 years	37 (24.0)	24 (16.7)	10 (33.3)	9 (30.0)	69 (21.8)	67 (21.6)
Race, *n* (%)						
White	123 (79.9)	116 (80.6)	24 (80.0)	24 (80.0)	253 (80.1)	248 (80.0)
Black/African American	28 (18.2)	23 (16.0)	4 (13.3)	5 (16.7)	53 (16.8)	53 (17.1)
Asian	2 (1.3)	4 (2.8)	1 (3.3)	1 (3.3)	7 (2.2)	7 (2.3)
American Indian/Alaska native	0	1 (0.7)	0	0	1 (0.3)	1 (0.3)
Other	1 (0.6)	0	1 (3.3)	0	2 (0.6)	1 (0.3)
Ethnicity, *n* (%)						
Hispanic/Latino	33 (21.4)	24 (16.7)	8 (26.7)	6 (20.0)	65 (20.6)	61 (19.7)
Not Hispanic/Latino	121 (78.6)	120 (83.3)	22 (73.3)	24 (80.0)	251 (79.4)	249 (80.3)
Body mass index (kg/m^2^), mean (SD)	28.75 (6.65)	29.96 (7.10)	31.25 (8.49)	31.64 (10.98)	29.56 (7.08)	29.70 (7.45)
OAB characteristics						
No. of patients	154	144	30	30	341	336
Duration of OAB (months), mean (SD)	81.85 (74.34)	75.11 (99.13)	74.06 (84.98)	67.16 (59.96)	76.98 (86.66)	77.57 (84.62)
Type of OAB, *n* (%)						
Urgency incontinence only	65 (42.2)	55 (38.2)	12 (40.0)	14 (46.7)	139 (40.8)	140 (41.7)
Mixed stress/urgency incontinence with urgency as predominant factor	53 (34.4)	50 (34.7)	10 (33.3)	8 (26.7)	116 (34.0)	112 (33.3)
Frequency/urgency without incontinence	36 (23.4)	39 (27.1)	8 (26.7)	8 (26.7)	86 (25.2)	84 (25.0)
Number of episodes/24 h, mean (SD)						
Incontinence	3.15 (4.22)	3.19 (4.62)	3.80 (5.25)	3.74 (5.10)	2.71 (4.25) [*n* = 336]	2.67 (4.18) [*n* = 334]
Urgency incontinence	2.86 (4.19)	2.77 (4.31)	3.71 (5.28)	3.54 (4.96)	2.34 (4.02) [*n* = 336]	2.27 (3.95) [*n* = 334]
Micturitions	11.25 (2.64)	11.65 (3.75)	12.77 (2.68)	11.81 (2.52)	10.34 (3.37) [*n* = 336]	10.08 (3.86) [*n* = 334]
Urgency (grade 3 or 4)	5.34 (4.11)	5.65 (4.73)	6.26 (4.54)	5.93 (4.34)	4.39 (4.31) [*n* = 336]	4.23 (4.46) [*n* = 334]
Nocturia	1.64 (1.04)	1.52 (0.97)	2.26 (1.05)	1.60 (1.18)	1.62 (1.04) [*n* = 277]	1.47 (1.00) [*n* = 280]
Incontinent patients at baseline of period 1, *n* (%)						
Wet	117 (76.0)	98 (68.1)	24 (80.0)	22 (73.3)	250 (73.3)	241 (71.7)
Dry	37 (24.0)	46 (31.9)	6 (20.0)	8 (26.7)	91 (26.7)	95 (28.3)
Previous non-drug treatment, *n* (%)						
Yes	6 (3.9)	6 (4.2)	1 (3.3)	3 (10.0)	11 (3.2)	17 (5.1)
No	148 (96.1)	138 (95.8)	29 (96.7)	27 (90.0)	330 (96.8)	319 (94.9)

*OAB* Overactive bladder

^a^Patients with the same treatment in two different periods (sequences M/M and T/T) are counted once

### Efficacy results

**Fig. 3:**
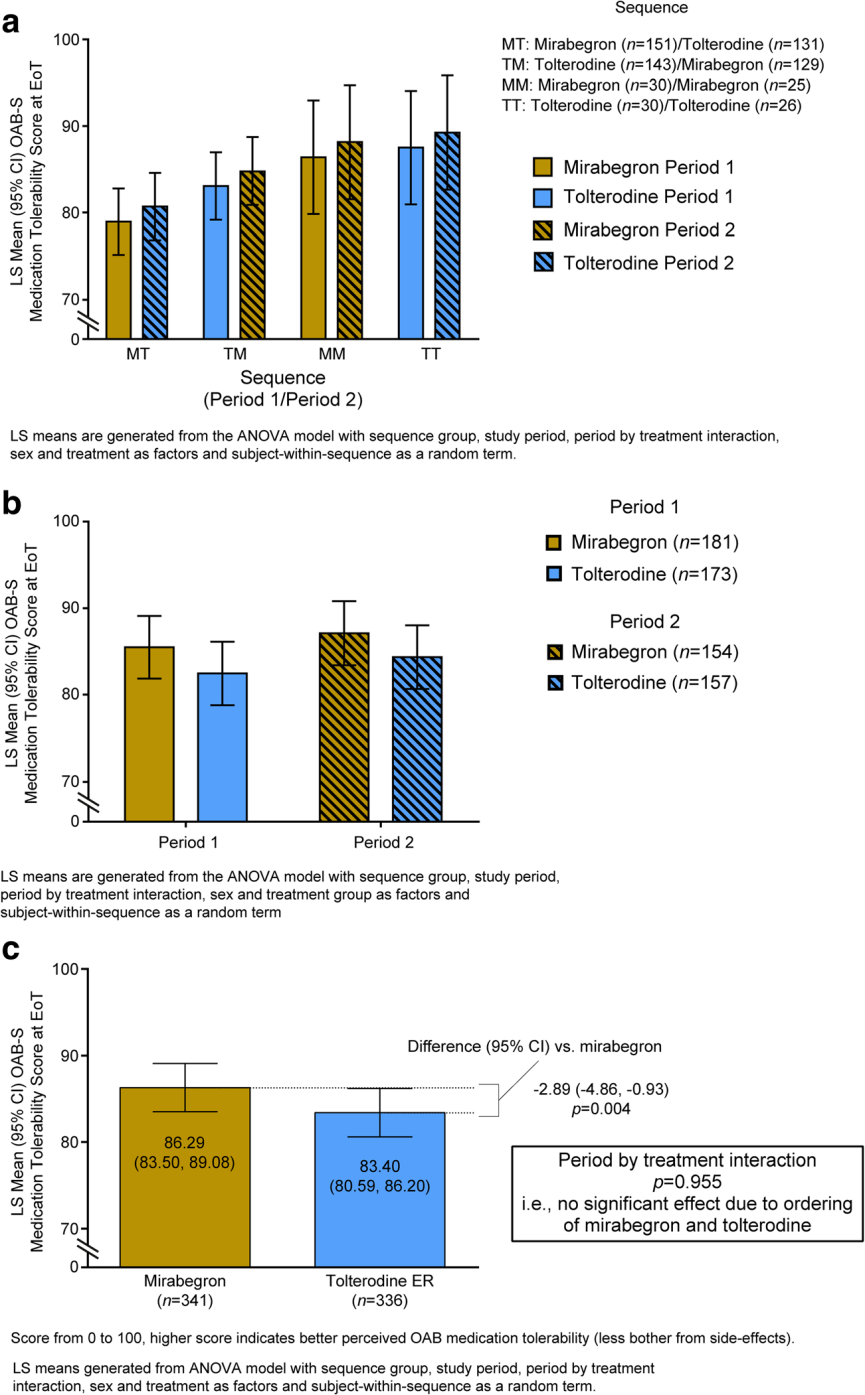
Mean (95% CI) OAB-S Medication Tolerability scores at end of treatment in the full analysis set: **a** by sequence, difference in period; **b** within period, difference in treatment; **c** overall treatment difference (primary endpoint)

Mean OAB-S Medication Tolerability scores were higher in period 2 for all sequences in the FAS (within-sequence analysis; Fig. 3a). The mean (95% CI) OAB-S Medication Tolerability scores were higher for mirabegron in both periods (period 1, 85.48 [81.85, 89.11]; period 2, 87.10 [83.39, 90.81]) than for tolterodine ER (period 1, 82.46 [78.80, 86.12]; period 2, 84.33 [80.65–88.01; within-period analysis, Fig. 3b). The period-by-treatment interaction, testing if the relationship between the OAB-S Medication Tolerability scores for tolterodine and mirabegron differed between the two treatment periods, was not statistically significant (*p* = 0.955); therefore, sequence (i.e., whether patients received mirabegron first or second) did not significantly affect the mean OAB-S Medication Tolerability scores, thus enabling direct comparison of treatments. In the T/M sequence group, OAB-S Medication Tolerability scores in period 1 were slightly lower than in the complete patient group (patients who entered both treatment periods), indicating that patients dropping out during period 1 (i.e., while receiving tolterodine ER in period 1) had a lower OAB-S Medication Tolerability score on average than patients who proceeded to period 2 (i.e., those who received mirabegron in period 1).

For the primary efficacy endpoint, the mean [95% CI] OAB-S Medication Tolerability scores were significantly higher in patients receiving mirabegron (86.29 [83.50, 89.08]) than in those receiving tolterodine ER (83.40 [80.59, 86.20]), representing a treatment difference in tolerability of −2.89 [−4.86, −0.93]; *p* = 0.004; Fig. 3c). For the secondary outcome of preference, 69.8% of patients receving the M/T and T/M sequences and 72.5% the M/M and T/T sequences reported a preference for either period. Among patients receiving both sequences (M/T and T/M), 48.3% preferred mirabegron and 51.7% preferred tolterodine ER (*p* = 0.77, not signficant). The percentage of patients reporting a strong preference was higher for mirabegron (70.6%) than for tolterodine ER (63.7%; not tested for significance). More patients selected the reason for their preference as “better treatment” (mirabegron 83.5% vs. tolterodine ER 89.0%) than selected “tolerated better” (mirabegron 24.7% vs. tolterodine ER 18.7%). However, patients were able to select more than one option. A slightly higher percentage of physicians had a strong preference for mirabegron (57.1%) than tolterodine ER (53.6%; not tested for significance).

At EoT, the majority of patients did not experience side effects as measured in terms of the individual components of the OAB-S Medication Tolerability score. The only exception was dry mouth, which was reported by 56.5% of patients during tolterodine ER treatment (vs. 44.5% during mirabegron treatment; Table 2). During tolterodine ER treatment more than half of patients who experienced dry mouth regarded it as bothersome (“a lot”, “moderately” or “somewhat”; Table 2).

**Table 2:** Analysis of individual components of the OAB-S Medication Tolerability score at end of treatment in the full analysis set

Side effect	Treatment	Total number of patients^a^	Did not have side effects, *n* (%)	Had side effects, *n* (%)				
				Bothered a lot	Bothered moderately	Bothered somewhat	Bothered little	Did not bother
Constipation	Mirabegron	335	233 (69.6)	11 (3.3)	12 (3.6)	20 (6.0)	40 (11.9)	19 (5.7)
	Tolterodine	330	227 (68.8)	10 (3.0)	11 (3.3)	28 (8.5)	37 (11.2)	17 (5.2)
Dry mouth	Mirabegron	336	190 (56.5)	18 (5.4)	21 (6.3)	27 (8.0)	59 (17.6)	21 (6.3)
	Tolterodine	330	147 (44.5)	46 (13.9)	28 (8.5)	23 (7.0)	65 (19.7)	21 (6.4)
Drowsiness	Mirabegron	336	200 (59.5)	12 (3.6)	21 (6.3)	32 (9.5)	46 (13.7)	25 (7.4)
	Tolterodine	330	201 (60.9)	21 (6.4)	18 (5.5)	25 (7.6)	40 (12.1)	25 (7.6)
Headache	Mirabegron	335	228 (68.1)	12 (3.6)	11 (3.3)	21 (6.3)	42 (12.5)	21 (6.3)
	Tolterodine	330	221 (67.0)	10 (3.0)	16 (4.8)	18 (5.5)	42 (12.7)	23 (7.0)
Nausea	Mirabegron	335	262 (78.2)	3 (0.9)	9 (2.7)	8 (2.4)	26 (7.8)	27 (8.1)
	Tolterodine	330	252 (76.4)	6 (1.8)	9 (2.7)	8 (2.4)	20 (6.1)	35 (10.6)
Blurred vision	Mirabegron	335	238 (71.0)	10 (3.0)	14 (4.2)	10 (3.0)	42 (12.5)	21 (6.3)
	Tolterodine	330	233 (70.6)	4 (1.2)	12 (3.6)	16 (4.8)	38 (11.5)	27 (8.2)

^a^Total number of patients at each visit per sequence and treatment

Improvements in the OAB-S Medication Tolerability score at EoT were more evident in women, patients aged ≥65 years, and in patients without baseline incontinence, and improvement was greater with mirabegron treatment than with tolterodine ER treatment (Supplementary Fig. 1). Specifically, in the gender subgroup analysis, mean OAB-S Medication Tolerability scores among both women and men were higher with mirabegron treatment (LS mean 84.14 for women, 88.40 for men) than with tolterodine ER treatment (LS mean 80.86 for women, 86.49 for men). The estimated improvement in mean [95% CI] OAB-S Medication Tolerability scores was greater among women (−3.28 [−5.62, −0.94) than among men (−1.91 [−5.49, 1.66]).

In the post hoc analysis of patient preference, men and patients aged ≥65 years were more likely to prefer mirabegron, whereas women and younger patients (<65 years) were more likely to prefer tolterodine ER. Baseline incontinence status did not appear to influence treatment preference (Supplementary Fig. 2). There were no differences between treatments in bladder diary variables at EoT and no significant effects of sequence on daily incontinence episodes and micturition frequency (Table 3). Among incontinent patients, the percentages of respondents achieving zero incontinence episodes at EoT with mirabegron and tolterodine ER treatment were 45.9% and 45.5%, respectively, and the percentages achieving a ≥50% reduction in incontinence episodes were 64.6% and 69.1%, respectively.

**Table 3:** Changes from baseline to end of treatment (EoT) in bladder diary variables

Variable	Population	Mirabegron				Tolterodine					Period-by-treatment interaction *p* value
		No. of patients	Baseline, mean (SE)	EoT, mean (SE)	Adjusted change from baseline to EoT, mean (SE), [95% CI]	No. of patients	Baseline, mean (SE)	EoT, mean (SE)	Adjusted change from baseline to EoT, mean (SE), [95% CI]	Adjusted difference vs. mirabegron, mean (SE) [95% CI]	
Incontinence episodes/24h	FAS-I	248	3.70 (0.29) [*n* = 245]	2.07 (0.25) [*n*=246]	–1.51 (0.19) [−1.89, −1.13]	240	3.69 (0.30) [*n*=238]	2.02 (0.23) [*n*=235]	−1.46 (0.19) (−1.84, −1.07)	0.05 (0.224) [−0.39, 0.49]	0.971
Number of micturitions/24h	FAS	341	10.34 (0.18) [*n*=336]	8.25 (0.18) [*n*=336]	–2.06 (0.19) [−2.44, −1.68]	336	10.08 (0.21) [*n*=334]	8.13 (0.19) [*n*=329]	−1.95 (0.20) [−2.33, −1.57]	0.11 (0.20) [−0.28, 0.50]	0.211
Urgency incontinence episodes/24h	FAS-I	248	3.20 (0.28) [*n*=245]	1.61 (0.22) [*n*=246]	–1.42 (0.20) [−1.81, −1.02]	240	3.16 (0.28) [*n*=238]	1.55 (0.21) [*n*=235]	−1.37 (0.20) [−1.76, −0.97]	0.05 (0.20) [−0.35, 0.45]	
Number of urgency episodes (PPIUS grade 3 or 4)/24h	FAS	341	4.39 (0.24) [*n*=336]	2.14 (0.19) [*n*=336]	–2.26 (0.19) [−2.64, −1.88]	336	4.23 (0.24) [*n*=334]	2.13 (0.19) [*n*=329]	−2.13 (0.19) [−2.51, −1.75]	0.13 (0.17) [−0.20, 0.46]	
Number of nocturia episodes/24h	FAS	341	1.62 (0.06) [*n*=277]	1.27 (0.06) [*n*=272]	–0.26 (0.06) (−0.38, −0.14)	336	1.47 (0.06) [*n*=280]	1.22 (0.05) [*n*=273]	−0.25 (0.06) (−0.37, −0.13)	0.01 (0.07) [−0.13, 0.15]	

*FAS* Full analysis set, *FAS-I* Full analysis set–incontinence

### Safety results

The overall percentages of TEAEs and serious TEAEs, respectively, were 47.0% and 0.9% with mirabegron and 51.7% and 2.5% with tolterodine ER (Table 4; Supplementary Table 2). TEAEs were more frequent in period 1 across all treatment sequences. The most common TEAEs were dry mouth (9.1% with mirabegron, 16.3% with tolterodine ER), constipation (5.6% and 6.2%, respectively) and headache (5.6% and 5.8%, respectively). Significant differences in favor of mirabegron were observed for anticholinergic TEAEs (20.4% and 27.4%, respectively; *p* = 0.042) and gastrointestinal disorders (14.7% and 22.5%, respectively; *p* = 0.015; Table 4). At EoT, increases in systolic and diastolic blood pressure from baseline were on average <1 mmHg for mirabegron and tolterodine ER and similar between treatments. Pulse rate increased on average by approximately 1 bpm and 2 bpm with mirabegron and tolterodine ER, respectively.

**Table 4:** Overall treatment-emergent adverse events (TEAEs), most common TEAEs (≥5% of patients in any treatment group) and TEAEs of special interest in the safety analysis set

TEAE	Number of patients (%)^b^		*p* value^c^
	Mirabegron (*n* = 319)	Tolterodine ER (*n* = 325)	
Any TEAE	150 (47.0)	168 (51.7)	–
Drug-related TEAE	89 (27.9)	111 (34.2)	–
Deaths	0	0	–
Serious TEAE	3 (0.9)	8 (2.5)	–
Drug-related serious adverse event^a^	2 (0.6)	0	–
TEAEs leading to permanent discontinuation of study drug	15 (4.7)	20 (6.2)	–
Drug-related TEAEs leading to permanent discontinuation of study drug^a^	12 (3.8)	12 (3.7)	–
Serious TEAEs leading to permanent discontinuation of study drug	0	5 (1.5)	–
Drug-related serious TEAEs leading to permanent discontinuation of study drug^a^	0	0	–
Most Common TEAEs (by Preferred Term)			
Dry mouth	29 (9.1)	53 (16.3)	–
Constipation	18 (5.6)	20 (6.2)	–
Headache	18 (5.6)	19 (5.8)	–
TEAEs of special Interest (by System Organ Class and Preferred Term)			
Common anticholinergic TEAEs	65 (20.4)	89 (27.4)	0.042
Gastrointestinal disorders	47 (14.7)	73 (22.5)	0.015
Dry mouth	29 (9.1)	53 (16.3)	–
Constipation	18 (5.6)	20 (6.2)	–
Nausea	6 (1.9)	8 (2.5)	–
Nervous system disorders	20 (6.3)	29 (8.9)	0.235
Headache	18 (5.6)	19 (5.8)	–
Somnolence	4 (1.3)	10 (3.1)	–
Eye disorders	12 (3.8)	11 (3.4)	0.835
Vision blurred	12 (3.8)	11 (3.4)	–
Respiratory, thoracic and mediastinal disorders	2 (0.6)	0	0.245
Dry throat	2 (0.6)	0	–
Cardiovascular TEAEs	3 (0.9)	5 (1.5)	0.725
Cardiac disorders	3 (0.9)	5 (1.5)	0.725
Atrial fibrillation	2 (0.6)	1 (0.3)	–
Tachycardia	1 (0.3)	2 (0.6)	–
Palpitations	0	2 (0.6)	–
Vascular disorders	11 (3.4)	9 (2.8)	0.656
Hypertension	11 (3.4)	9 (2.8)	–
Urinary retention TEAEs	1 (0.3)	1 (0.3)	1.000
Renal and urinary disorders	1 (0.3)	1 (0.3)	1.000
Urinary retention	1 (0.3)	1 (0.3)	1.000
Urinary tract infections	12 (3.8)	17 (5.2)	–
Hypersensitivity	2 (0.6)	5 (1.5)	–
Glaucoma	0	0	–

The data are presented as number (%) of patients

^a^Possible or probable drug-related event, as assessed by the investigator, or records where relationship was missing

^b^If a patient reported a TEAE for the same treatment in two different periods (sequences MM/TT), then that patient was counted once

^c^ *p* values were calculated for the common anticholinergic side effects, cardiovascular events and urinary retention events, and were based on Fisher’s exact test

## Discussion

OAB becomes problematic for patients when daily QoL is affected. This emphasizes the importance of measuring symptom improvement from the patient’s perspective, as well as measuring changes in bladder diary parameters, particularly as objective improvements in urinary frequency and incontinence episodes do not always translate into improved QoL [9]. It is also evident that significant improvements in QoL are not always reflected in satisfaction and persistence with therapy [16]. Patient satisfaction associated with medication tolerability may be a meaningful outcome that differentiates oral pharmacotherapies for OAB.

Mirabegron was associated with statistically significantly higher medication tolerability scores than tolterodine ER, particularly in women, patients aged ≥65 years, and patients without baseline incontinence. Contrary to our hypothesis, however, improved tolerability of mirabegron was not associated with a medication preference. It should be noted that tolerability is a balance between efficacy and adverse events, and the majority of patients in this trial gave perceived better efficacy as the reason for their preference. OAB-S Medication Tolerability scores in period 1 were generally slightly lower than the scores in patients who completed both periods (complete cases), and in particular were lower among patients receiving tolterodine ER in period 1. Hence, patient discontinuation during period 1 due to tolerability would not have been accounted for in the preference analysis because preference was only measured at the end of period 2. Moreover, the Likert scale used to evaluate preference has not been validated in OAB trials and may not have been sufficiently sensitive to detect differences in preference. The reason why tolerability did not influence treatment preference, and the observed differences in treatment preference by sex and age warrant further investigation. The observed tolerability benefit with mirabegron, however, was corroborated by treatment differences in anticholinergic adverse events, most notably dry mouth, which was extremely bothersome, occurring at almost three times the rate among patients receiving tolterodine ER than among those receiving mirabegron. Improvement in micturition diary variables was comparable between treatments. Almost half of patients (about 45%), both those receiving tolterodine ER and those receiving mirabegron, achieved complete resolution of incontinence, while the majority (>60%) achieved a reduction in daily incontinence episodes by at least 50%.

>Both treatments were well tolerated. The statistically significant difference in favor of mirabegron for anticholinergic TEAEs, and more specifically, gastrointestinal disorders, was predominantly because of the difference in the frequency of dry mouth between patients receiving mirabegron (9.1%) and those receiving tolterodine ER (16.3%). Dry mouth was assessed in two ways: the first via unsolicited spontaneous reporting as an adverse event, as done in all pharmaceutical trials, and the second as a specific response item of the OAB-S Medication Tolerability scale. The difference in the methods of capture, spontaneous versus solicited, likely explains the large discrepancy in the rates of dry mouth reported in this study (i.e., 9.1% and 43.5% for mirabegron vs 16.3% and 55.5% for tolterodine ER) between the two methodologies. However, both methods were directionally consistent with substantially more reports of dry mouth among patients receiving tolterodine ER. There were no clinically meaningful increases in blood pressure among patients receiving mirabegron or tolterodine ER and the magnitude of the increases was similar to those reported in other studies [17, 18]. The higher incidence of TEAEs in period 1 across all sequences suggests that adverse events might be experienced shortly after starting treatment or that patients became tolerant and reported adverse events less frequently in period 2. The magnitude of improvements in OAB symptoms, response rates and incidence of TEAEs are consistent with those reported with mirabegron and tolterodine ER monotherapy in phase III studies [4, 7, 19–21].

This is the first late-phase OAB clinical trial to utilize a crossover design and explore patient satisfaction using the OAB-S questionnaire. The crossover design is more efficient at determining within-patient differences since patients serve as their own matched control. The inclusion of sequences in which patients received the same drug twice allowed unbiased estimation of treatment effects irrespective of carry-over effects. The study had adequate power to detect small differences in OAB-S Medication Tolerability scores. The inclusion of a treatment-naive population provided an unbiased assessment of tolerability; this cohort would be expected to be less tolerant of side effects than previously treated patients. The potential carry-over effects and 10% discontinuation rate between treatment periods may have impaired the detection of a sequencing effect on efficacy outcomes. The mirabegron dose increase reflects a clinically plausible regimen since the recommended starting dose in North America is 25 mg and shows good efficacy at 4 weeks, but efficacy is not maximized until about 8 weeks [22].

### Conclusions

The use of mirabegron for the treatment of OAB in treatment-naive patients was associated with a statistically significantly higher OAB-S Medication Tolerability score than the use of tolterodine ER. Treatment preference and objective improvements in OAB symptoms were comparable between the treatments. Both drugs were well tolerated. However, anticholinergic side effects were higher with tolterodine ER. Further studies should evaluate additional domains of satisfaction with OAB therapies to help differentiate treatments and tailor therapy according to patient priorities and lifestyle, and increase satisfaction and persistence.

## Electronic supplementary material

Supplementary Fig. 1 Mean (95% CI) OAB-S Medication Tolerability scores by sex (**a**), age (**b**) and baseline incontinence (**c**) in the full analysis set (GIF 50 kb)

(GIF 49 kb)

(GIF 54 kb)

High resolution image (TIFF 61 kb)

High resolution image (TIFF 61 kb)

High resolution image (TIFF 67 kb)

Supplementary Fig. 2 Patient preference by sex and age (**a**) and baseline incontinence status (**b**) in the full analysis set–preference/no preference population (GIF 73 kb)

(GIF 57 kb)

High resolution image (TIFF 105 kb)

High resolution image (TIFF 89 kb)

Supplementary file 1 Randomization and blinding (DOCX 12 kb)

Supplementary Table 1 Inclusion and exclusion criteria (DOCX 17 kb)

Supplementary Table 2 Overall TEAEs, most common TEAEs (≥5% of patients in any treatment group) and TEAEs of special interest (SAF) by gender (DOCX 13 kb)
