# Supplementary figures and images for: A prospective, double-blind, randomized, two-period crossover, multicenter study to evaluate tolerability and patient preference between mirabegron and tolterodine in patients with overactive bladder (PREFER study)
Source: Int Urogynecol J. 2017 Jun 15;29(2):273–83. doi: 10.1007/s00192-017-3377-5 (PMC5780540; doi:10.1007/s00192-017-3377-5)

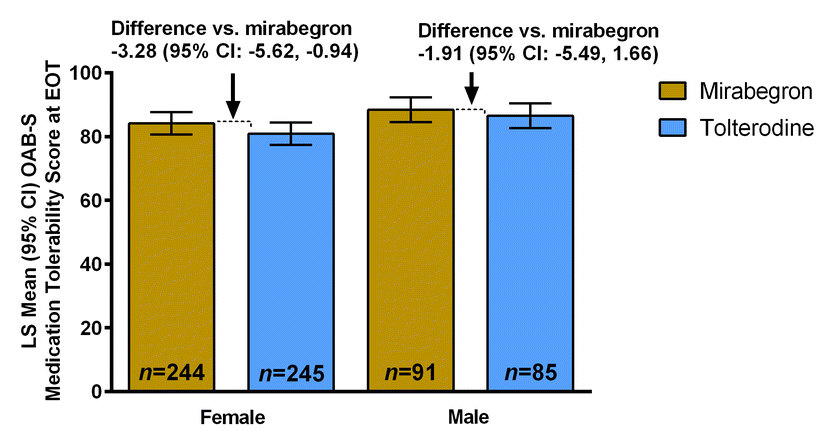

Supplement: Supplementary file 1 — Mean (95% CI) OAB-S Medication Tolerability scores by sex (a), age (b) and baseline incontinence (c) in the full analysis set (GIF 50 kb) [file 192_2017_3377_Fig4_ESM.gif]

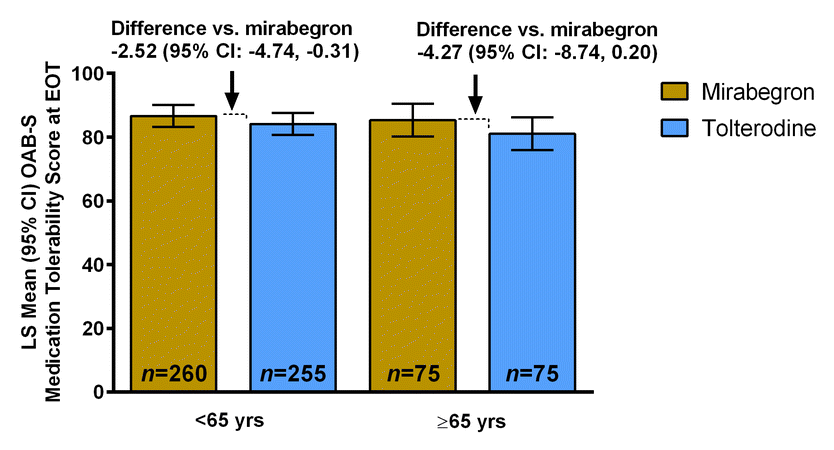

Supplement: Supplementary file 2 — (GIF 49 kb) [file 192_2017_3377_Fig5_ESM.gif]

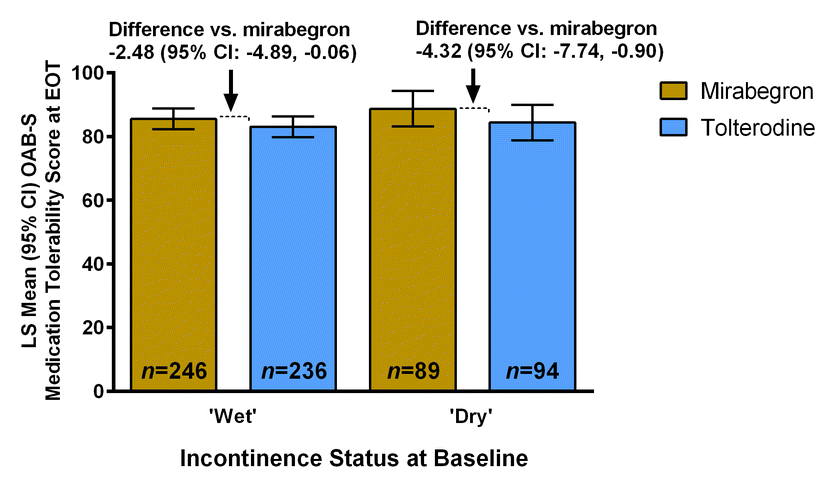

Supplement: Supplementary file 3 — (GIF 54 kb) [file 192_2017_3377_Fig6_ESM.gif]

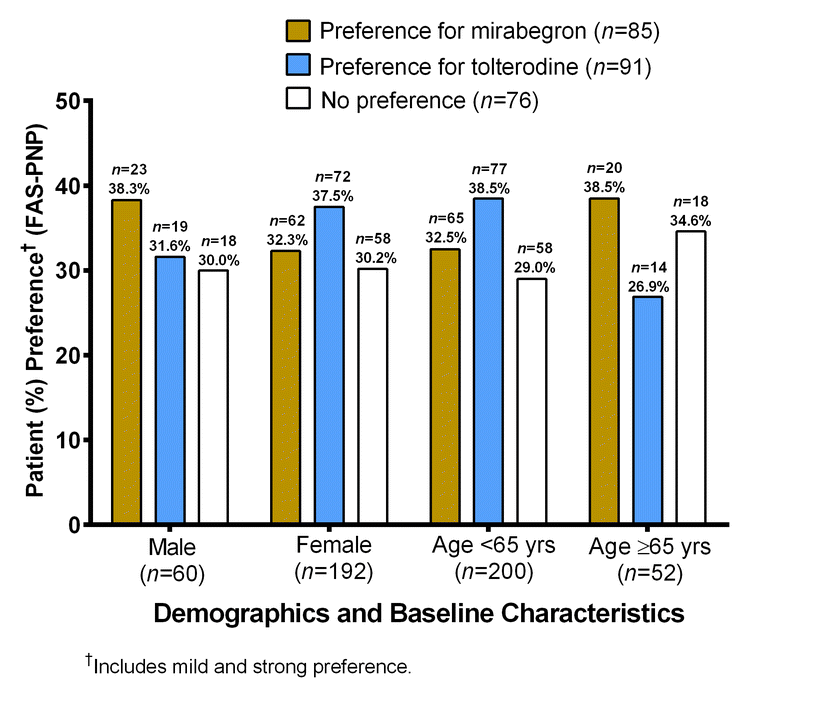

Supplement: Supplementary file 7 — Patient preference by sex and age (a) and baseline incontinence status (b) in the full analysis set–preference/no preference population (GIF 73 kb) [file 192_2017_3377_Fig7_ESM.gif]

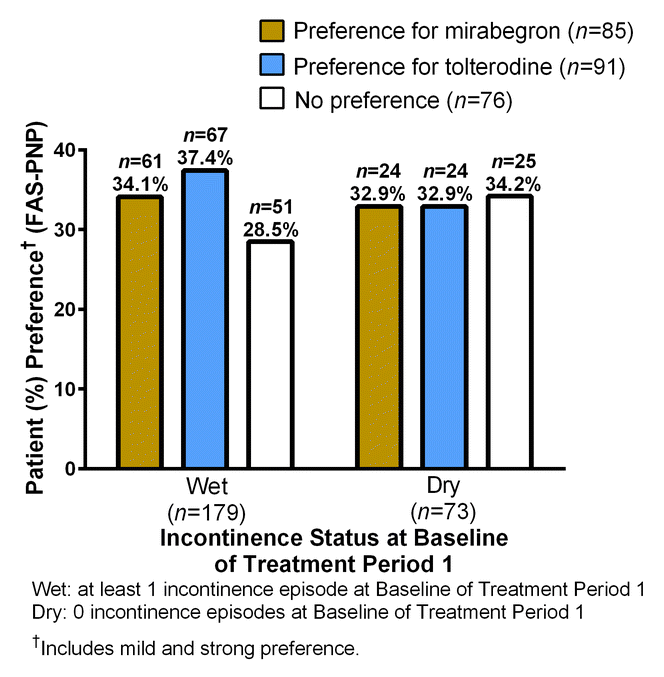

Supplement: Supplementary file 8 — (GIF 57 kb) [file 192_2017_3377_Fig8_ESM.gif]
