# Supplementary material for: A prospective, double-blind, randomized, two-period crossover, multicenter study to evaluate tolerability and patient preference between mirabegron and tolterodine in patients with overactive bladder (PREFER study)
Source: Int Urogynecol J. 2017 Jun 15;29(2):273–83. doi: 10.1007/s00192-017-3377-5 (PMC5780540; doi:10.1007/s00192-017-3377-5)
Supplement: Supplementary file 11 — Randomization and blinding (DOCX 12 kb) [file 192_2017_3377_MOESM1_ESM.docx]

**Supplementary file 1.** Randomization and blinding

*Randomization*

After a patient signed informed consent, a patient number was assigned by the Investigator or designee utilizing web-based Interactive Response Technology (IRT). Patients who met all the inclusion and none of the exclusion criteria were randomly assigned at visit 2 (week 0) to receive 1 of 4 sequences (mirabegron/tolterodine, tolterodine/mirabegron, mirabegron/mirabegron, or tolterodine/tolterodine) using a 5:5:1:1 randomization schedule generated by the Astellas Global Data Science department, or designee. Randomized treatment was obtained by the Investigator or designee utilizing web-based IRT. Study drug assignment remained blinded to all staff. Study drug was packaged in kits preprinted with a kit number. Study drug kits for periods 1 and 2 were assigned at the week 0 and week 10 visits, respectively. The kit number assigned to the patient was noted in the eCRF for study drug.

If a patient was assigned a patient number but did not receive study drug, the patient number was not used again.

*Blinding*

The Investigator, study coordinator(s), patients, sponsor and sponsor’s representatives were blinded to the identity of the randomized drug assignment.

Study drug assignment was revealed only for reasons relating to the patient’s safety or when critical therapeutic decisions were contingent on knowing the assigned study drug. Except in the most pressing circumstances, a decision to break the blind was to be discussed with the sponsor or sponsor’s designee. Withdrawal of a patient from the study was not a sufficient reason to break the blind. The sponsor could break the treatment code for patients who experienced a Suspected Unexpected Serious Adverse Reaction, in order to determine if the individual case or a group of cases required expedited regulatory reporting.
