# Supplementary material for: A prospective, double-blind, randomized, two-period crossover, multicenter study to evaluate tolerability and patient preference between mirabegron and tolterodine in patients with overactive bladder (PREFER study)
Source: Int Urogynecol J. 2017 Jun 15;29(2):273–83. doi: 10.1007/s00192-017-3377-5 (PMC5780540; doi:10.1007/s00192-017-3377-5)
Supplement: Supplementary file 12 — Inclusion and exclusion criteria (DOCX 17 kb) [file 192_2017_3377_MOESM2_ESM.docx]

**Supplementary Table 1** Inclusion and exclusion criteria

| **Inclusion criteria** | **Exclusion criteria** |
| --- | --- |
| ***At Screening (Visit 1)*** | |
| - IRB-/Independent Ethics Committee-approved written informed consent from the patient prior to any study‑related procedures - Male or female patient aged ≥18 years - Patient was willing and able to complete the bladder diary and questionnaires correctly - Patient had symptoms of OAB (urinary frequency and urgency with or without incontinence) for ≥3 months prior to screening - Patient must have been treatment-naïve to pharmaceutical agents for OAB - Female patient must have been either:   - Postmenopausal (at least 1 year without any menses in the absence of other plausible etiology prior to screening) or   - Documented surgically sterile or status posthysterectomy (at least 1 month prior to screening) or   - If of childbearing potential, must have had a negative serum pregnancy test at screening and must have used a highly effective method of birth control, which included the established use of oral, injected or implanted hormonal methods of contraception, placement of an intrauterine device (IUD) or intrauterine system (IUS) or barrier methods of contraception: condom or occlusive cap (diaphragm or cervical/vault caps) with spermicidal foam/gel/film/cream/suppository. Birth control must have been practiced from screening and throughout the study period and for 30 days after the final dose of study drug. - Male and female patients and their spouses/partners who were of childbearing potential must have been using a highly effective method of birth control, which included established use of oral, injected or implanted hormonal methods of contraception, placement of an IUD or IUS. Birth control must have been practiced from screening and continued throughout the study and for 30 days after the final study drug administration - Female patient must have not donated ova starting at screening and throughout the study period and for 30 days after the final study drug administration - Male patient must have not donated sperm starting at screening and throughout the study period and for at least 30 days after final study drug administration - Patient agreed not to participate in another interventional study from the time of screening until the final study visit | - Female patient who was lactating or was intending to breastfeed during the study period and for 30 days after the final study visit - In the opinion of the investigator, the patient had clinically significant bladder outlet obstruction posing a risk of urinary retention - Patient had significant stress incontinence or mixed stress/urgency incontinence where stress was the predominant factor as determined by the investigator - Patient had evidence of urinary tract infection (UTI) (positive leukocyte esterase was confirmed with a urine culture greater than 100000 colony forming units/mL) as assessed at screening. The patient could have been rescreened after successful treatment of the UTI (confirmed by a laboratory result of negative urine culture) - Patient had a neurological cause for detrusor overactivity (e.g., neurogenic bladder, diabetic neuropathy or systemic or central neurological disease such as multiple sclerosis and Parkinson’s disease) - Patient had an indwelling catheter or practiced intermittent self-catheterization - Patient had a chronic inflammatory condition such as interstitial cystitis, bladder stones, previous pelvic radiation therapy or previous or current malignant disease of the pelvic organs (i.e., within the confines of the pelvis including the bladder and rectum in both sexes and the uterus, ovaries and fallopian tubes in females; or of the lower gastrointestinal tract) - Patient had uncontrolled narrow angle glaucoma, urinary or gastric retention, severe colitis ulcerosa, toxic megacolon, myasthenia gravis, polio or any other medical condition which, in the opinion of the investigator, made the use of anticholinergics contraindicated - Patient had received intravesical injection in the past 12 months with botulinum toxin, resiniferatoxin or capsaicin - Patient had received invasive treatment including electro-stimulation therapy (e.g., percutaneous tibial nerve stimulation) - Patient was receiving a bladder training program or pelvic floor exercises which started or had changed less than 30 days prior to screening - Patient had hepatic impairment defined as Child-Pugh Class A, B or C - Patient had severe renal impairment defined as creatinine clearance less than 30 mL/min, end-stage renal disease or undergoing dialysis - Patient had severe uncontrolled hypertension (sitting systolic blood pressure (SBP) ≥180 mm Hg and/or diastolic blood pressure (DBP) ≥110 mm Hg - Patient had evidence of QT prolongation on ECG (QT interval corrected for heart rate using Fridericia’s formula (QTcF) >450 msec for males, >470 msec for females) or a known history of QT prolongation - Patient had a clinically significant ECG abnormality, as determined by the investigator - Patient had a serum creatinine >150 μmol/L, or aspartate aminotransferase (AST) or alanine aminotransferase (ALT) >2 x upper limit of normal (ULN), or γ-glutamyl transpeptidase (GGT) >3 x ULN and considered clinically significant by the investigator - Patient had a hypersensitivity to any components of mirabegron, other β-AR agonists, tolterodine or other antimuscarinic agents or any of the inactive ingredients - Patient had any clinically significant condition, which in the opinion of the investigator makes the patient unsuitable for study participation - Patient had been treated with an experimental device within 30 days or received an investigational agent within 30 days prior to screening - Patient had a concurrent malignancy or history of any malignancy (within the past 5 years), except nonmetastatic basal or squamous cell carcinoma of the skin that had been treated successfully - Patients with current history of alcohol and/or drug abuse - Patient was using prohibited medications which could not be stopped safely during the period defined - Patient was involved in the conduct of the study as an employee of the Astellas group, third party associated with the study or the study site team - Patient had stopped, started or changed the dose of a restricted medication within the last 30 days prior to screening |
| ***At Randomization (Visit 2)*** | |
| - Inclusion criteria of Visit 1 are met - ≥ 3 episodes of urgency (grade 3 or 4) recorded during 3-day bladder diary - Average ≥ 8 micturitions/day recorded during 3-day bladder diary | - Exclusion criteria at Visit 1 fulfilled |
