# Supplementary material for: A prospective, double-blind, randomized, two-period crossover, multicenter study to evaluate tolerability and patient preference between mirabegron and tolterodine in patients with overactive bladder (PREFER study)
Source: Int Urogynecol J. 2017 Jun 15;29(2):273–83. doi: 10.1007/s00192-017-3377-5 (PMC5780540; doi:10.1007/s00192-017-3377-5)
Supplement: Supplementary file 13 — Overall TEAEs, most common TEAEs (≥5% of patients in any treatment group) and TEAEs of special interest (SAF) by gender (DOCX 13 kb) [file 192_2017_3377_MOESM3_ESM.docx]

**Supplementary Table II.** Overall TEAEs, most common TEAEs (≥5% of patients in any treatment group) and TEAEs of special interest (SAF) by gender

|  |  | **Number of patients (%)*** | |  |
| --- | --- | --- | --- | --- |
| **Overview of TEAEs** | Mirabegron  (*n*=319) | | Tolterodine ER  (*n*=325) | |
|  | Women  (*n*=234) | Men  (*n*=85) | Women  (*n*=246) | Men  (*n*=79) |
| ***TEAEs of Special Interest***  **System Organ Class**  **Preferred Term** |  |  |  |  |
| *Common Anticholinergic TEAEs* |  |  |  |  |
| Gastrointestinal disorders | 52 (22.2) | 9 (10.6) | 68 (27.6) | 17 (21.5) |
| Dry mouth | 26 (11.1) | 3 (3.5) | 41 (16.7) | 12 (15.2) |
| Constipation | 14 (6.0) | 4 (4.7) | 18 (7.3) | 2 (2.5) |
| Nausea | 6 (2.6) | 0 | 6 (2.4) | 2 (2.5) |
| Nervous system disorders | 26 (11.1) | 3 (3.5) | 23 (9.3) | 8 (10.1) |
| Headache | 16 (6.8) | 2 (2.4) | 14 (5.7) | 5 (6.3) |
| Somnolence |  |  |  |  |
| Eye disorders | 13 (5.6) | 6 (7.1) | 11 (4.5) | 3 (3.8) |
| Vision blurred | 8 (3.4) | 4 (4.7) | 9 (3.7) | 2 (2.5) |
| Respiratory, thoracic and mediastinal disorders | 10 (4.3) | 2 (2.4) | 10 (4.1) | 3 (3.8) |
| Dry throat | 1 (0.4) | 1 (1.2) | 0 | 0 |
| *Cardiovascular TEAEs* |  |  |  |  |
| Cardiac disorders | 3 (1.3) | 1 (1.2) | 4 (1.6) | 1 (1.3) |
| Atrial fibrillation | 1 (0.4) | 1 (1.2) | 0 | 1 (1.3) |
| Tachycardia | 1 (0.4) | 0 | 2 (0.8) | 0 |
| Palpitations | 0 | 0 | 2 (0.8) | 0 |
| Vascular disorders | 7 (3.0) | 5 (5.9) | 9 (3.7) | 2 (2.5) |
| Hypertension | 6 (2.6) | 5 (5.9) | 7 (2.8) | 2 (2.5) |
| *Urinary Retention TEAEs* |  |  |  |  |
| Renal and urinary disorders | 6 (2.6) | 4 (4.7) | 9 (3.7) | 6 (7.6) |
| Urinary retention | 1 (0.4) | 0 | 1 (0.4) | 0 |

*If a patient reported a TEAE for the same treatment in two different periods (sequences MM/TT), then that patient was counted once

***p* values were calculated for the common anticholinergic side effects, cardiovascular events and urinary retention events and were based on Fisher's exact test

†Possible or probable, as assessed by the investigator, or records where relationship was missing

*ER*, extended release, *SAF*, Safety Analysis Set, *TEAE*, treatment-emergent adverse event
